# Perspective: Preservation of coherence in photophysical processes

**DOI:** 10.1063/1.5079265

**Published:** 2018-12-26

**Authors:** Theis I. Sølling, Klaus B. Møller

**Affiliations:** 1 Center for Integrative Petroleum Research, College of Petroleum and Geosciences, King Fahad University of Petroleum and Minerals, Dhahran 31261, Saudi Arabia; 2 Department of Chemistry, Technical University of Denmark, Kemitorvet 207, 2800 Kgs. Lyngby, Denmark

## Abstract

Coherence is one of the most important phenomena in ultrafast sciences. We give our perspective on the terminology, observation, and preservation of coherence in photophysical processes with some glimpses to the past and some looking-head to what may pave the way for scaling one of the last bastions in ultrafast science, namely, that of mode specific chemistry where it will be possible to break any specific bond by tailoring the pulse, an accomplishment that obviously would be the dream of any chemist.

Zewail's centennial paper on NaI truly highlighted the importance of being able to observe recurrences in the transient data that result from an optical pump-probe experiment.^1^ In the NaI case, the data unequivocally showed the real-time motion of two atoms connected by a chemical bond—a stretching motion that clearly leads to a transition back and forth between two distinct electronic states.^2^ Since then, observations of oscillating signals that evolve on the femtosecond timescale have shown up in experimental studies of myriads of systems, and the underlying structural dynamics have been ascribed to a variety of processes that are more or less complex in nature.^3–5^ The common denominator in all the experiments, where the observable oscillates periodically, is that there is always something “extra” to be said about the nuclear motions involved in the photoinduced processes in those cases, especially when exposed to theoretical treatment.^6^ These considerations are not new and have been discussed in great detail in two very comprehensive reviews with combined roughly 300 references.^7,8^ The focus of the present perspective is on how nuclei sometimes keep moving coherently even after processes that are usually thought to randomize the energy and so not as much on coherence as a phenomenon but rather on its preservation.

Oscillating signals are often referred to as being “coherent.” While this is in reality a mis-denomination, the oscillations are a result of nuclei moving coherently and the recurring experimental signal assists in the interpretation of how.^3^ For nuclei to be observed to move coherently via an oscillatory signal, the internal vibrational energy—and phases—cannot be randomly distributed because if it was the observable that could not depend systematically on time and the temporal evolution of the signal would in essence be random. The presence of externally disturbing factors, such as, for example, solvation and diverging processes, may result in randomization of the energy and phases. As a result, it is less likely to observe an oscillatory signal from processes that are exposed to external perturbation. On a timescale faster than that of randomization of the energy and phases, it is usually envisioned that only a single or at least very few combinations of the molecular degrees of freedom are excited. This can give rise to a distinguishable change in the molecular structure as a function of time. Thus, with appropriate temporal and spatial (phase) resolutions, one would be able to observe oscillatory signals and in principle be able to translate the observation into real-time structural information.

This is, however, not always the case in optical pump-probe experiments; such an observation requires a change in absorption propensity as a function of the active nuclear motion. That is, two different nuclear positions should give rise to a significantly different number of absorbed photons. This scenario is quite easily accomplished in an experiment where the nuclear motion directly couples two electronic states, with the electronic nature of the initial state being distinctively different from that of the final state, just as was the case for NaI.^1^ An example of this could play out in the photophysics of aliphatic amines.^9–11^ Here, the four lowest lying states are associated with the excitation of a lone pair electron to an orbital with a high principal quantum number, i.e., a Rydberg orbital, to generate a so-called Rydberg state. Such Rydberg excited molecules in essence behave as if they have an ionic core with a weakly interacting electron in the distance. Thus, the initial motion that is induced after the excitation to a Rydberg state is the one that equilibrates from the structure of the neutral to a structure resembling that of an ionized species. In the amine case, this essentially means a change from a tetrahedral geometry in the nitrogen to a planar one (Fig. 1).^9^

**FIG. 1.**
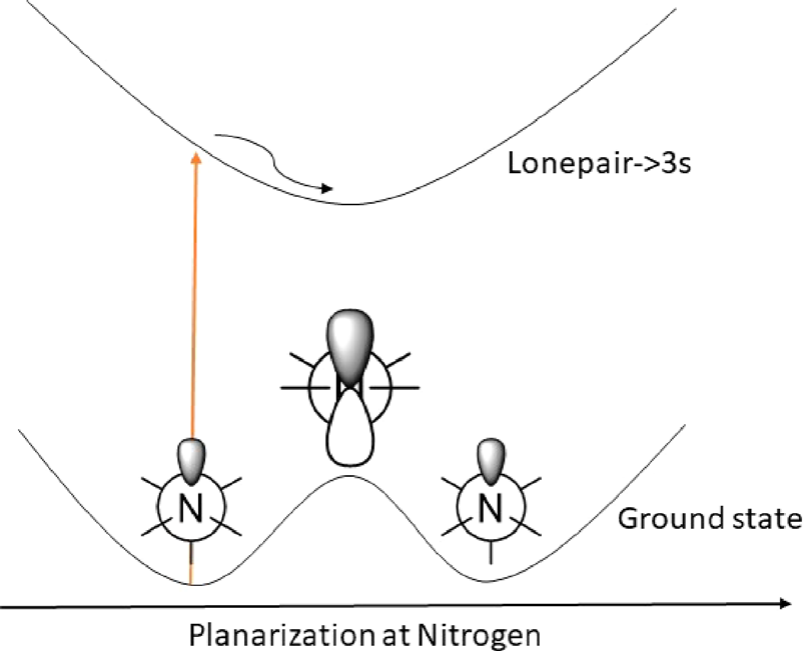
Illustration of how the most favorable configuration changes in the nitrogen when a lonepair electron is excited to a Rydberg orbital.

This planarization motion can in principle be followed in real-time if a state exists such that the excitation energy changes as a function of the pyramidalization angle. All lonepair to Rydberg excitations inevitable will have very similar characteristics as defined by the ionic core with an electron missing at the site of the lonepair and the associated diffuse electron. Accordingly, the energy change as a function of the pyramidalization angle is the same in all cases—or to put it in another way—the potential energy surfaces are if not parallel then at least almost parallel. Thus, a strategy that involves real time visualization (via an oscillating absorbance) of planarization at the nitrogen by excitation of the Rydberg electron to a higher-lying Rydberg orbital is deemed to be challenging [Fig. 2(a)]. The probe excitation should involve either excitation from an occupied orbital other than the lonepair (e.g., a σ-orbital) or from the lonepair to a virtual orbital other than a Rydberg (e.g., a σ*-orbital) [Fig. 2(b)].

**FIG. 2.**
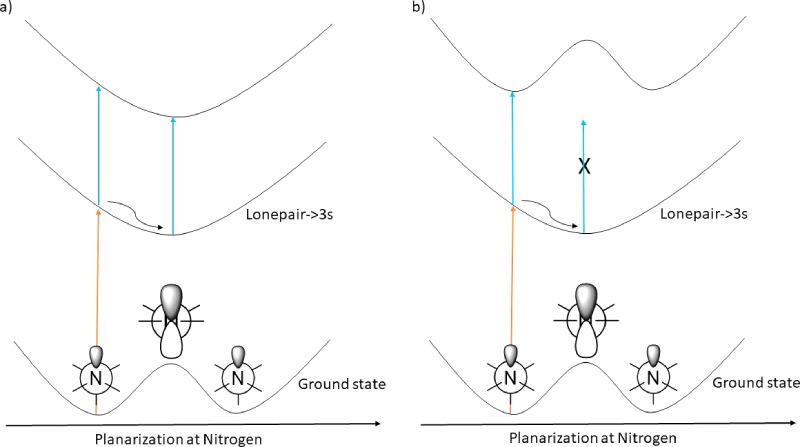
Illustration of the absorption schemes and thus potential ways of observing real time molecular motion in amines in a Rydberg state (a) and as an alternative, a valence state (b).

This would result in absorption that changes periodically as a function of angle as long as the planarization is the only degree of freedom that is involved initially. When the energy dissipates, the observed absorption will be time independent because some molecules absorb the light efficiently whereas others do not. The description presented above directly in terms of absorption is applicable in transient absorption experiments. However, the considerations are identical when the observable is a property other than absorption. In, for example, time resolved mass spectrometry experiments or time resolved photoelectron experiments, which take place in the gas phase which means that no disturbing solvent effects are in play, the propensity for forming ions and photoelectrons by interaction with the probe also depends on how well the probe is absorbed by the excited state species that is generated by the pump.^9^ A significant difference is, however, that the end state is an ion which means that the potential energy surface of the end state and of, for example, a Rydberg state will in theory always be close to parallel. This does not apply to a ionization out of valence states, and indeed oscillating signals have been observed in experiments with the ionizing probe. A classical example is that of NaI where it is fairly obvious that the observed oscillations result from the stretching motion.^1^ Oscillating signals that result from experiments that rely on ionization of more complex systems have been observed by mass spectrometric detection and by integrating the photoelectron signal in a manner that reveals subtle differences in kinetic energy as a function of time delay.^12,13^ The most challenging aspect of addressing oscillating signals from pump probe experiments on complex systems is to convert the observed frequencies associated with the oscillating signals into the actual temporally resolved molecular structure. Specifically, the active mode is in a bath of the remaining degrees of freedom which eventually will channel the energy away from the initially excite degrees of freedom.

The rate of internal conversion has often times been described in terms of statics; Fermi's golden rule, for example, involves the density of states in the reactant as well as in the receiver states. The fact that complex systems encompass many degrees of freedom should prevent the continuous preservation of vibrational energy in a limited number of the available degrees of freedom subsequent to internal conversion or intersystem crossing. For NaI, it was doable because here really only a single degree of freedom is present. However, recently, this was exactly what has been found in a series of experiments; the transition from one state to another renders the energy localized in the exact same degree of freedom as was originally activated.^14^ The conclusion arises from the observation of oscillatory signals associated with nuclear motions in electronic states that were not initially involved in the excitation process.

A very evident case of such a preservation phenomenon has been observed for N-methylmorpholine by Weber and co-workers.^15^ In an elegant pump-probe photoelectron experiment which involved a Rydberg pump excitation best described as nitrogen lonepair −> 3p, the 3p photoelectron trace that results from ionization by the probe dies out within the first picosecond. This is exactly the time it takes for the 3s photoelectron signal to appear; thus, in this manner, it is shown that a transition from a 3p to the 3s orbital is in play. Moreover, the signal associated with the 3p state reveals a clear oscillatory component; the motion that is required for the ground state molecular structure to adapt to the new electronic environment in the Rydberg state is planarization at the nitrogen, and so, the oscillatory nature of the signal is taken to be a result of the initial motion on the 3p excited state surface. In this particular case, the experiment very clearly shows that this motion remains the only one active in the 3s state after the transition because the oscillatory component of the signal persists. It seems that there are some very stringent conditions for the preservation of coherence; the investigation of the very similar albeit unsymmetrical N-methylisomorpholine did not show preservation of coherence, and so, it seems that symmetry restrictions are what make the molecules follow a specific path that does not allow for the internal energy to transition into other degrees of freedom during the internal conversion process.^16^ Such an effect of the preservation of coherence might be applied: For example, by paying attention to the symmetry of a system, it might be possible to tune the excited state dynamics of a molecule. A subtle control could be utilised in the design of solar cells, for example, by designing a molecule in such a way as to prevent motions in normal modes that are able to couple surfaces. This would increase the lifetime of the excited state and potentially increase the efficiency of energy transfer.

Ionic open-shell systems can show a similar type of behavior: We have observed that excited-state azobenzene radical cations preserve, in transition from D_1_ to D_0_, the tortional motion that is initiated when the molecular structure adapts to the new electronic configuration on the D_1_ surface.^17^ Just as for the morpholines above, the preservation of coherence was revealed by conducting experiments on two different isomers. In the azobenzene case, the molecules in play were the cis- and the trans-isomers. The oscillatory data were found to be exactly phase shifted by π but are otherwise nearly the same. This strongly indicates that the two isomers proceed through a common structure and the observation allows for reconstruction of the potential energy surface (which is authenticated by calculations) in Fig. 3 where it can be seen that the motion in play is the cis-trans isomerization motion involving the phenyl groups.

**FIG. 3.**
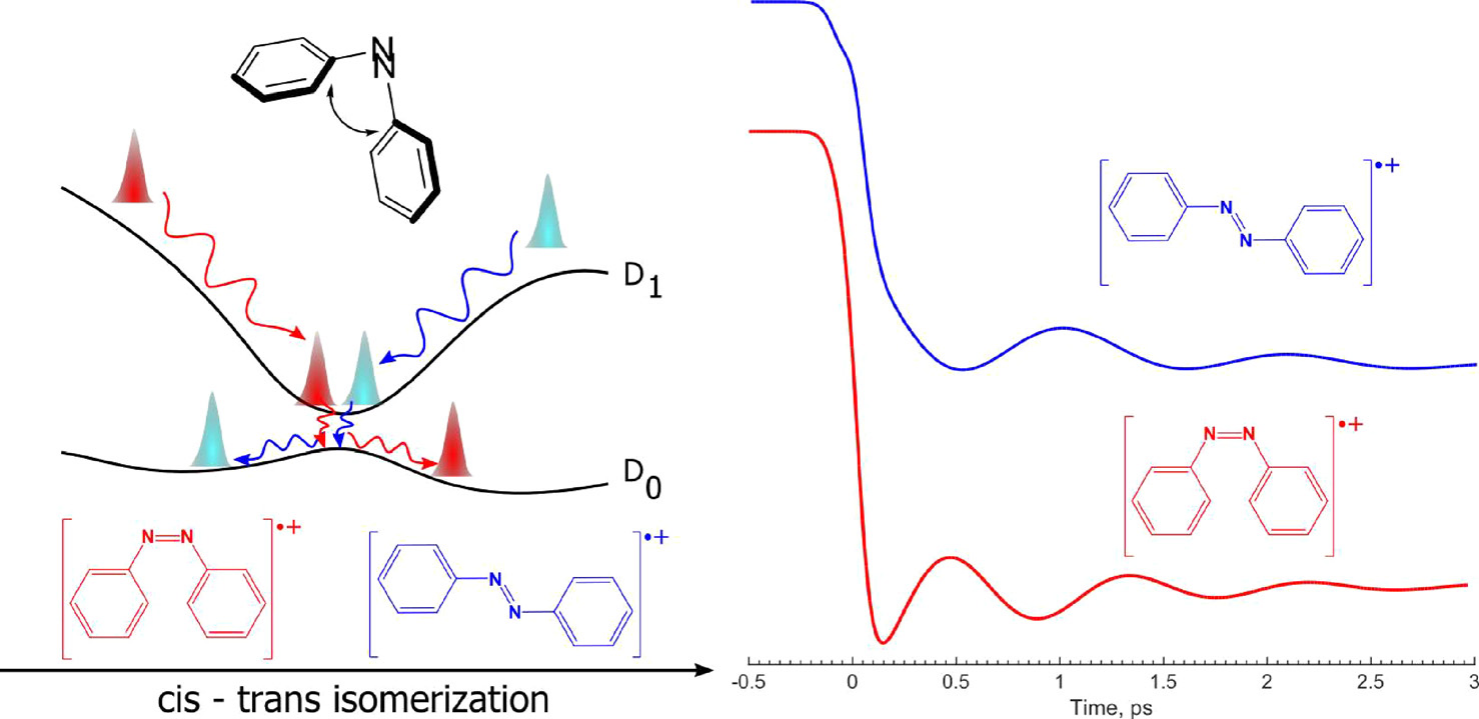
The mechanism internal conversion of internal conversion in the ionized open-shell azobenzene system where the involvement of two complementary isomers clearly showed that a common intermediate connects the D_1_ and D_0_ states.

Like for the morpholine study, the use of complementary isomers is reflected in the experimental results, which enables a more direct visualization of the nuclear motions that are involved in initiating or even driving the photophysics, in systems as complex as azobenzene.

Chergui and co-workers^18^ have recently studied the all-time classic (see Levi *et al.*^19^ and references therein) tetrakis(μ-pyrophosphito)diplatinate(II) [Pt_2_(μ-P_2_O_5_H_2_)_4_]^4−^, also known as Pt(pop), in a transient absorption experiment. In contrast to the azobenzene and morpholine experiments, the Pt(pop) experiments were carried out in the solution phase. Thus, apart from the structural complexity of the system, the solvent shell encapsulating the anion might have been anticipated to delocalize the excitation energy and prevent the internal energy to be localized for a long time. Nevertheless, the transient absorption signal is clearly oscillatory. The data show that a transition from the initial singlet state to the first excited triplet takes place and that the vibrational excitation is preserved in the same manner as for azobenzene and N-methylmorpholine. So regardless of the fact that an intersystem crossing is in play and regards of the potential solvent effects induced by acetonitrile the energy still stays put. It seems that the common denominator is having a rigid system with a high degree of symmetry and that this is key to prevent the excitation energy from escaping in an ocean of randomness. In this context, a solvent may actually prevent dephasing by enforcing rigidity through steric hindrance.^20^ The authors speculate that what matters for preservation of vibrational coherence to be in play is the solvation environment; acetonitrile accelerates the intersystem crossing which then prevents the delocalization of the vibrational excitation energy. This could certainly be yet a contributing factor in addition to rigidity and symmetry.

So, where is all of this leading? Quite a few summarizing remarks could be made, and directions for further studies of preservation of coherence are plentiful. The findings are reiterating that internal conversion (and also intersystem crossing) processes are dynamically driven processes in the sense that the motion out of the Franck-Condon region takes the molecule in the “just right” direction to transition from one state to another.^14^ This may be seen as a catch-22 because if a transition was not possible via “direction” by the initially active motions, it would not be ultrafast and no longer on radar of the community. It nevertheless seems to be the case that for all the excitations to high lying states, there is always an ultrafast pathway out; moreover, in the cases shown here, it is proven that the lower lying states are accessed via the initially activated degrees of freedom. So, perhaps, the right question to ask is why such a pathway always seems to exist? A question that may be addressed with more precise and mode-specific excitation and extension of probing methods beyond optical spectroscopy.^21^

All three examples of preservation of “coherence” which have been highlighted in this perspective address one or another form of possible means of control; symmetry, rigidity, and solvation. This may pave the way for scaling one of the last bastions in ultrafast science, namely, that of mode specific chemistry where it will be possible to break any specific bond by tailoring the pulse, an accomplishment that obviously would be the dream of any chemist.
